# Leptin: Is It Thermogenic?

**DOI:** 10.1210/endrev/bnz016

**Published:** 2019-11-27

**Authors:** Alexander W Fischer, Barbara Cannon, Jan Nedergaard

**Affiliations:** 1 Department of Molecular Biosciences, The Wenner-Gren Institute, The Arrhenius Laboratories F3, Stockholm University, Stockholm, Sweden; 2 Department of Biochemistry and Molecular Cell Biology, University Medical Center Hamburg-Eppendorf, Hamburg, Germany

**Keywords:** leptin, brown adipose tissue, thermogenesis, *ob/ob* mouse, energy expenditure, body temperature

## Abstract

Animals that lack the hormone leptin become grossly obese, purportedly for 2 reasons: increased food intake and decreased energy expenditure (thermogenesis). This review examines the experimental evidence for the thermogenesis component. Analysis of the data available led us to conclude that the reports indicating hypometabolism in the leptin-deficient *ob/ob* mice (as well as in the leptin-receptor-deficient *db/db* mice and *fa/fa* rats) derive from a misleading calculation artefact resulting from expression of energy expenditure per gram of body weight and not per intact organism. Correspondingly, the body weight-reducing effects of leptin are not augmented by enhanced thermogenesis. Congruent with this, there is no evidence that the *ob/ob* mouse demonstrates atrophied brown adipose tissue or diminished levels of total UCP1 mRNA or protein when the *ob* mutation is studied on the inbred C57BL/6 mouse background, but a reduced sympathetic nerve activity is observed. On the outbred “Aston” mouse background, brown adipose tissue atrophy is seen, but whether this is of quantitative significance for the development of obesity has not been demonstrated. We conclude that leptin is not a thermogenic hormone. Rather, leptin has effects on body temperature regulation, by opposing torpor bouts and by shifting thermoregulatory thresholds. The central pathways behind these effects are largely unexplored.

## Graphical Abstract

**Graphical Abstract:**
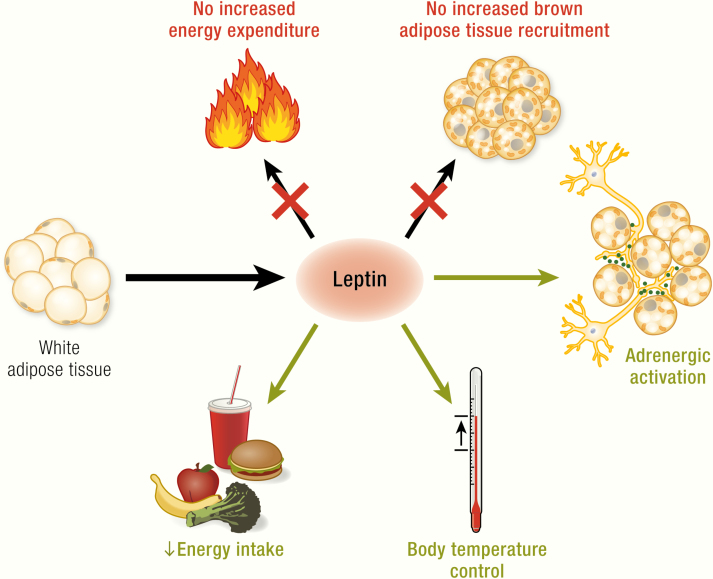

**ESSENTIAL POINTS**

- Leptin counteracts obesity by reducing energy intake; additionally a thermogenic effect has been suggested to further promote the maintenance of a lean phenotype
- The evidence for a thermogenic function of leptin is based only on normalization of energy expenditure to body weight
- The lower body temperature in the leptin-deficient *ob/ob* mice is not due to low thermogenesis but to a lower threshold for body temperature regulation
- In brown adipose tissue, thermogenic parameters appear lower in *ob/ob* mice than in wildtype when expressed in a normalized way (e.g. per mg protein) – but total thermogenic capacity is not lowered; the tissue thus shows pseudo-atrophy
- The lack of thermogenic effects of leptin are analogous in studies of the leptin-deficient *ob/ob* mice, the leptin receptor-deficient *db/db* mice and *fa/fa* (Zucker) rats, and leptin-deficient humans
- Thus, the anti-obesity effects of leptin through hypophagia are not augmented through increased thermogenesis

eptin is generally considered to affect body energetics in 2 cooperating ways, both counteracting obesity: decreasing appetite as well as increasing the combustion of food (i.e., increasing thermogenesis) (Fig. 1). Although there is a plethora of review articles on the role of leptin and leptin deficiency on regulation of food intake (and insulin sensitivity, reproduction, immunity etc.) (e.g., (1–7)), there is no comprehensive study on the significance of leptin for thermoregulation, especially with respect to the role of leptin in the control of brown adipose tissue (BAT) activity. This review attempts to fill this gap.

**Figure 1.**
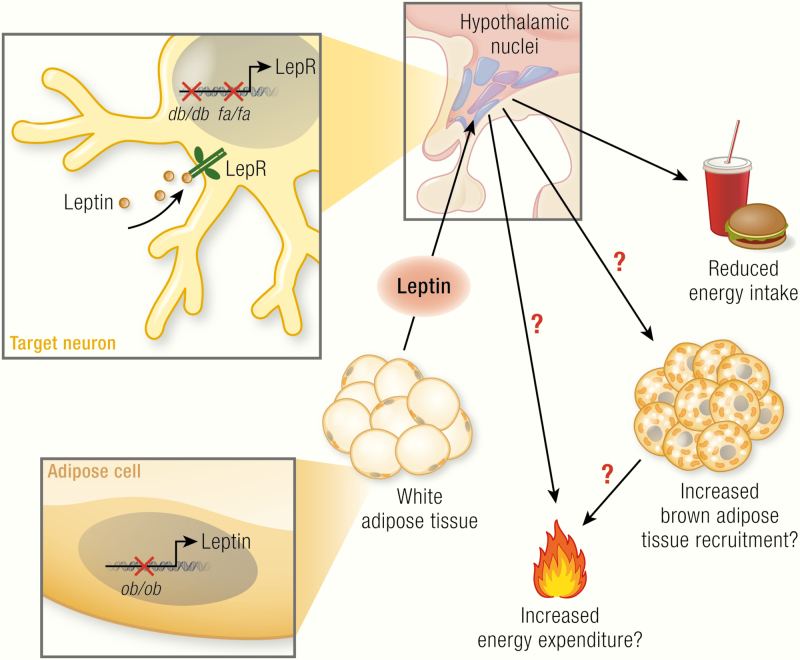
The general picture of leptin action. Leptin, encoded by the *ob* gene, is expressed in adipocytes (mainly in white adipocytes); its expression is positively correlated with fat mass and regulated in a fasting/feeding-dependent manner. Leptin is secreted and via the circulation reaches its target neurons in hypothalamic nuclei (colored circles, here unspecified) that express the long isoform of the leptin receptor (LepRb, green). Mice carrying the *ob* mutation lack functional leptin; animals carrying the *db* mutation (mice) or *fa* mutation (rats) lack functional LepRb. Leptin action in the hypothalamus activates anorexigenic pathways and is discussed to also trigger mechanisms affecting brown adipose tissue activity and, mainly through this, energy expenditure. The latter two effects are still under debate and are those analyzed in the present review. In the adipocytes: blue circles, nuclei; dark-brown structures mitochondria; yellow circles, lipid droplets; in the neurons: beige circles, leptin.

Knowledge on the thermogenic role of leptin has been obtained from observations on animals that lack leptin function and from direct observations of leptin effects. We therefore first analyze relevant data from the *ob/ob* mouse (also known as Lep^ob^/Lep^ob^), which lacks the ability to produce leptin, and from the *db/db* mouse and *fa/fa* rat, which lacks the leptin receptor. We then examine the effects of leptin treatment in both leptin-deficient mice and wild-type mice, as well as the effects of leptin in humans. We conclude that leptin is a pyrexic hormone (increases the regulated body temperature) but that it is not thermogenic, at least not in the standard mouse models used, and that the obesity in the *ob/ob* mice thus evolves without reinforcement from decreased thermogenesis.

## What Is the *ob/ob* Mouse?

In 1949, researchers at the Jackson Laboratories observed a spontaneous mutation in a mouse colony that led to excessive hyperphagia and weight gains. Because of the massive obesity of these mice, the mutation was called obese (*ob/ob*) (8). These obese mice further suffered from hyperglycemia, hyperinsulinemia, and infertility (6,9–11). A recessive mutation in a gene located on chromosome 6 was analyzed to be the cause of the phenotypes observed (9,12). Parabiosis experiments revealed that *ob/ob* mice lack a circulating factor (6,9,13–15). In 1994, Friedman and colleagues identified this factor as a new hormone, leptin (16). In the *ob/ob* mouse, a C→T mutation in codon 105 of the leptin gene changes an arginine codon to a stop codon, resulting in expression of a truncated, nonfunctional form of leptin (16).

## The Genetic Background

*ob/ob* mice have to be bred heterozygously because homozygous animals are infertile. Thus, when +/*ob* mice are used, the resulting litters will consist of +/+, +/*ob*, or *ob/ob* animals. *ob/ob* mice can easily be identified by their rapid development of obesity, whereas +/*ob* and +/+ mice are phenotypically identical and normal wild-type, and are traditionally referred to as “lean littermates.”

An important factor influencing the effects of the *ob* mutation is the genetic background used. The *ob* mutation was initially discovered in a not fully characterized strain, the so-called V-stock in the Jackson Laboratories (8). Subsequently, it has been transferred to and maintained on the C57BL/6 background in the United States at Jackson Laboratories and in Scandinavia in Umeå. Whereas adult lean mice usually weigh about 25 g, *ob/ob* mice on the BL/6 background display body weights between ~40 g (17) and ~60 g (18). In England, the mutation was transferred to the not fully characterized outbred “Aston” strain. Because the Aston mice are outbred, they are generally heavier than the C57BL/6 mice, that, like most inbred strains, are stunted. Adult lean Aston mice generally display body weights between 30 g and 35 g and *ob/ob* mice about 70 to 100 g (19,20). The *ob* allele has also been transferred to FVB (21,22), Balb/c (23,24) and BL/Ks strains (10), but there is no thermogenesis-related information concerning these latter strains (except body temperature and cold tolerance in Balb/c, which will be discussed later). Thus, because nearly all experiments on thermoregulation have been performed on the C57BL/6 mouse, we first summarize the data obtained on this background, including results in Aston mice when they are similar. However, where discrepancies between the different strains exist, they will be discussed in a separate section on the Aston mice.

## Why Study the Effects of Leptin Deficiency on Thermoregulation?

The first observations of defective thermoregulation in leptin-deficient mice were made by Barrnett, Davis, and Mayer in the early 1950s (17,25). These researchers found that it could be useful to overcome the metabolic inertia of the adipose depots in the *ob/ob* mice by fasting the mice. This led to increased mobilization of fat stores and thus made the mice slimmer. They rationalized that placing the *ob/ob* mice under conditions that would increase their metabolism, such as exposure to cold temperatures, would lead to even larger effects on fat mobilization. They thus transferred 12 lean and 21 obese mice from 25°C into a 3°C cold environment. While the lean mice all endured the cold exposure without any problems, Mayer and Barrnett found to their surprise that 19 of the 21 *ob/ob* mice were dead after 3 hours in the cold (25). After this report, *ob/ob* mice have been considered to be severely cold intolerant. Numerous studies have since been designed to understand the underlying cause of this extreme phenotype. It is of importance to recognize here that such an experimental setting will mainly be able to detect defects in insulation and in muscle shivering thermogenesis, and not in non-shivering thermogenesis, which is only recruited after prolonged cold exposure.

Importantly, the inability of the *ob/ob* mice to produce sufficient heat to counteract the heat loss has not only been seen as a defect in thermogenesis itself. Particularly, the concept has been expanded to encompass the idea that this low level of thermogenesis (and perhaps a low metabolic rate in general) could promote the development of obesity (and consequently that leptin should in itself promote thermogenesis and thus promote weight loss) (Fig. 1) (26).

## Is Increased Heat Loss the Reason for the Cold Intolerance?

An inability to defend body temperature could be due to increased heat loss or to decreased heat production. It has been speculated that *ob/ob* mice may display increased heat loss in the cold, due to the greater surface-volume ratio caused by the obesity (17,27,28). However, already in 1954 (17), it was noted that gold-thioglucose-induced obese mice do not show such cold intolerance, despite having similar surface-volume ratios. Similarly, standard diet-induced obese mice are not cold intolerant (29–32).

**Figure 2.**
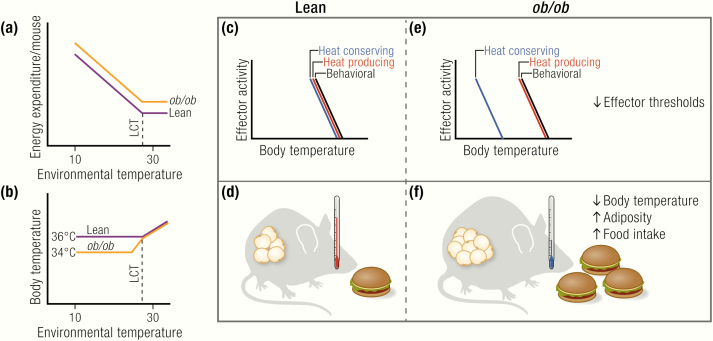
Are *ob/ob* mice hypothermic? (A) *ob/ob* mice have similar or even higher levels of energy expenditure than wild-type mice under thermoneutral conditions as well as under subthermoneutral conditions, at least on the C57BL/6 background. The limit of their thermoneutral zone, as indicated by the lower critical temperature (LCT), is similar in lean and *ob/ob*. (B) o*b/ob* mice have normal body temperature at thermoneutrality and defend a lower, but stable, body temperature when gradually exposed to subthermoneutral temperatures. (C, E) Differences in body temperature resulting from changes in the activation thresholds of thermoregulatory, but not behavioral, effectors. (D, F) Leptin-deficient *ob/ob* mice develop massive obesity because of excessive food intake, but not from reduced energy expenditure, which is similar or even higher than that in lean mice.

An increase in heat loss from a greater surface area or lower insulation can be quantified based on the cold-induced increase in energy expenditure observed in Scholander-type experiments (33) (Fig. 2A). A steeper increase in energy expenditure with an increased degree of cold is an indication of a lower insulative capacity, thereby increasing the need to produce heat to maintain euthermia. When such experiments have been performed in *ob/ob* mice, they have not supported the hypothesis of increased heat loss (20,27,34–36) (but see calculations on thermal conductance (27)).

In contrast to these surface-to-volume-based tenets, it is more generally supposed that increased obesity would rather protect against heat loss from an insulating effect of the fat layer. This general wisdom is, however, probably incorrect (34,37). The underlying cause for the massive drop in body temperature of the *ob/ob* mice upon acute cold exposure must thus be located elsewhere. One possibility that has been discussed is the low physical activity of the *ob/ob* mice (38,39), which may lead to reduced heat production. In agreement with this, *ob/ob* mice with reduced body weight from restricted feeding are more active (38,40). This means of limiting the body weight of the *ob/ob* mice by restricted feeding clearly improves their cold tolerance (38).

## *ob/ob* Mice Can Survive the Cold

The cold-induced drop in body temperature in acute cold (i.e., environmental temperature ~5°C) can be prevented by previous acclimation of the *ob/ob* mice to cool temperatures (12°C) for about 2 weeks (20).

Because the mice do not die when exposed to gradually decreasing temperatures (20,35), it would appear that the acute cold intolerance of the *ob/ob* mouse is mainly due to a difficulty for the mice to react fast enough and in a proper way to the decreased temperature (e.g., by decreasing heat loss and increasing thermogenesis). This observation is in itself surprising because such Scholander-type experiments usually involve exposing the animals to gradually decreasing temperatures over ~12 hours (i.e., a timeframe not long enough to induce any recruitment of heat-producing mechanisms). However, in these experiments, the mice have access to food and are thus not dependent on mobilization of their internal fat stores for heat production. The reason for the “slow reaction” of *ob/ob* mice to decreasing temperatures is not fully understood but reduced general adrenergic responsiveness in *ob/ob* mice would contribute to this phenotype (see later).

## *ob/ob* Mice Die in Acute Cold: What Happens at Other Temperatures?

*ob/ob* mice clearly become severely hypothermic when acutely exposed to cold temperatures (~5°C) (20,38,41–47). However, even at room temperature, these mice display about 1.5 to 2°C lower body temperatures than lean mice (20,27,28,35,38,45,47–54) (but see (38)).

Some groups also reported the body temperature of *ob/ob* mice to be lower in a thermoneutral environment of 28 to 30°C (18,41,42,50), whereas other groups have reported that the *ob/ob* body temperature at thermoneutrality was normal (20,27,35,46).

The overall interpretation of the existing data is that *ob/ob* mice display lower body temperatures when housed at subthermoneutral temperatures. The main question here is as to whether this is due to an inability of the mice to defend a higher body temperature (i.e., hypothermia) or from a change in the internal effector thresholds for body temperature regulation (that we refer to as anapyrexia) (Fig. 2BCE). That is to say, do the mice “want” (or “accept”) to be colder than their lean counterparts, or would they like to maintain a higher body temperature but are unable to do so?

## *ob/ob* Mice Are Anapyrexic

*ob/ob* mice display a diurnal rhythmicity in body temperature, even at colder temperatures, just as lean mice do. The body temperature of these mice is stable at a ~1.5 to 2°C lower level (18,35,41,50,51) (Fig. 2B). In the classical discourse on thermoregulation, in which a general “set point” of body temperature is implied, this may be interpreted as a change in the “defended” body temperature of the mice (i.e., they “want” to maintain a lower body temperature than their lean littermates) (Fig. 2CE). Within this type of model, a change in set point would be observable in all manifestations of thermoregulation.

One way to examine alterations in the defended body temperature (set point) in this model is to expose the animals to a thermogradient experiment, in which the animal can choose the desired environmental (floor) temperature in a metal tunnel (35,55). Mice defending a lower body temperature would be expected here to choose a colder environment to be able to dissipate the heat produced by their basal metabolism. Such experiments have, however, not revealed changes in the behaviorally controlled temperature preference of *ob/ob* mice (i.e., the *ob/ob* mice chose the same environmental temperature as wild-type mice) (35,56) (Fig. 2CE), but see (57), where lean mice display unexpectedly low preferred temperatures.

The seemingly surprising observations on behavioral thermoregulation in *ob/ob* mice may be interpreted differently if instead the threshold view is accepted (58,59). In this view, every thermo-effector has its own temperature threshold for activation, independent of other effectors. There is thus no body temperature set-point, although there appears to be one if these effector thresholds are close to one another. In this view, the reason that *ob/ob* mice display a reduced body temperature when they are exposed to subthermoneutral temperatures is that the threshold for the initiation of heat-conserving mechanisms is lower in the absence of leptin. The threshold for behaviorally regulated body temperature is, however, not shifted. Thus, when given the chance (e.g., in a temperature gradient), they will place themselves so that they display a normal wild-type-like body temperature (35,60) (Fig. 2CE).

## Does “Low Metabolism” Make the *ob/ob* Mouse Fat and Cold?

Although *ob/ob* mice thus seem to display a regulated, anapyrexic reduction in body temperature when being chronically exposed to subthermoneutral temperatures, the nature of the large and potentially lethal decrease in body temperature in *ob/ob* mice upon acute cold exposure has not yet been explained. Several hypotheses have been put forward, the most broadly held being the idea of “low metabolism” in these mice, leading to an inability to increase metabolism in the cold that will ultimately lead to the death of the animal. In this view, the low body temperature is thus hypothermia, not anapyrexia. The main support for such a hypothesis came from studies reporting lower energy expenditure rates in *ob/ob* mice as compared to lean controls. This, however, seems to be mainly based on (generally erroneous) ways of normalizing energy expenditure to body mass, an issue that is discussed later.

As implied previously, the excessive weight gain of *ob/ob* mice has been attributed not only to their hyperphagia, but also to changes in energy expenditure. The main rationale for this hypothesis is data derived from pair-feeding experiments, in which *ob/ob* mice pair-fed with lean mice still gained substantially more weight (61–64). This led to the assumption that “low metabolism” could be a causative factor for obesity. To analyze such a phenomenon, especially in animals such as *ob/ob* mice that show large alterations in body temperature in response to changes in environmental temperature and food availability, choosing the appropriate thermal environment is of paramount importance to exclude confounding effects caused by reductions in body temperature. This will be discussed later.

## Erroneous and Misleading Normalization of Energy Expenditure

An important issue when measuring energy expenditure is the question of whether to normalize the data or to express them per whole animal. If one, for example, wants to compare metabolic rates of animals of various sizes (e.g., mice and elephants), normalization to body mass (or to [body mass]^0.75^) can be a useful tool to correct for the different sizes of the animals examined.

However, when comparing lean and obese mice, this way of normalization to body weight will always result in misleading conclusions. In obese mice, the main compartment of weight gain is the increased fat mass. Although there is undoubtedly also a slight increase in lean mass (connective tissue, immune cells, cytoplasm of adipocytes, blood vessels, and increased muscle mass), the major part of the weight difference is caused by pure lipid. These lipids, just like the contents of a bottle of olive oil, are per se metabolically inert. They can of course be hydrolyzed and used in metabolism, but the fat itself is metabolically inert. Normalization to body weight will thus always underestimate the metabolic activity of obese mice because the energy expenditure will be divided by a higher body weight value partly consisting of an inert substance. Instead, expression per whole mouse or per lean body mass should be used, as repeatedly discussed (34,35,65–71). Also, plotting individual energy expenditure values with the corresponding lean mass, combined with analysis of covariance, has been suggested to be a useful tool to separate true effects on energy expenditure and effects confounded by alterations in lean mass (71). However, this method is not useful when the lean weight is very similar between individuals, as is often the case.

Indeed, in most of the studies claiming lower energy expenditure in *ob/ob* mice, normalization methods have been used. In (72), it is clear that, when expressed per mouse, basal oxygen consumption in *ob/ob* mice shows only a trend toward lower levels; only when expressed per gram body weight does it reach significance. In (28), normalized oxygen consumption (i.e., per gram body weight) in old obese mice is only half that of lean mice, but the body weight is doubled. The same is true for (52) and to a lesser extent for (73). Some studies report energy expenditure per mouse and per body weight for the same mice (36,54), clearly demonstrating that only when normalized to total body weight do *ob/ob* mice appear hypometabolic.

Thus, most studies show similar or even increased levels of energy expenditure in *ob/ob* mice based on unnormalized data expressed per mouse, either in free-moving (18,27,35,54,74,75) or restrained/anesthetized (18,19,70,76) animals. We thus conclude that *ob/ob* mice are not hypometabolic; they are probably even hypermetabolic (Fig. 2A), especially when considering the slightly lower lean mass in these animals.

## Pair-feeding Experiments Are Confounded by Initiation of the Energy-conserving Mechanism of Daily Torpor

Even when given the same amount of calories as the lean controls, *ob/ob* mice still become obese (pair-feeding experiments). This may at first sight be seen as convincing evidence for reduced metabolism in *ob/ob* mice (26). However, this interpretation is probably confounded because of the effects of the phenomenon of torpor.

Mice that experience an acute lack of food can enter daily torpor (77). In torpor, the body temperature is allowed to drop to near the environmental temperature; this normally takes place during daytime (the sleeping phase). Under normal animal house conditions, with ambient temperatures of about 20°C, body temperature can thus readily decrease to below 30°C, in a circadian pattern. A lower metabolism induced by the low body temperatures will then conserve energy.

Similarly, *ob/ob* mice that are pair-fed to wild-type mice will experience this as an acute lack of food and therefore be inclined to enter daily torpor (35,78–81). Consequently, they will also demonstrate decreased metabolism during the torpor bout. In pair-feeding experiments, the same amount of food that is sufficient to sustain normal metabolism in wild-type mice will be available to the *ob/ob* mice. These mice maintain, as indicated a lower total daily energy expenditure, because of the torpor bouts. Therefore, the energy not needed to maintain metabolism can be stored in these mice. This means that they become more obese than the wild-type, with the same food intake. It is close at hand to formulate this as demonstrating the existence of a leptin-dependent thermogenesis but, in reality, it is a demonstration of the effect of leptin to maintain normal body temperature.

Correspondingly, the decreasing leptin levels observed during starvation or food restriction are a necessary prerequisite for the initiation of torpor (82,83). Indeed, leptin prevents torpor induction (35,79,81,84–88); because this inhibitory factor is absent in *ob/ob* mice, they are especially susceptible to entering torpor (89,90).

An indication that it is the torpor as such that is responsible for the energy accumulation in the *ob/ob* mice is the effect of environmental temperature. The magnitude of the reduction in metabolism is larger in *ob/ob* mice at room temperature than at thermoneutrality (35), because the body temperature becomes lower. Consequently, the amount of extra energy that is stored as body fat in pair-fed *ob/ob* mice is larger at room temperature than at thermoneutrality (91).

Thus, that *ob/ob* mice become obese even when pair-fed to wild-type does not demonstrate a thermogenic effect of leptin but confirms the significance of leptin for body temperature regulation.

## Measurements at Thermoneutrality Reveal the True Effects of Metabolism on Obesity Development

When measuring basal metabolism in the mouse, and principally also in humans, the gold standard is to measure basal metabolic rate or resting metabolic rate in a thermoneutral environment (92–94). In such a setting, the individual is placed in an environment free from thermal stress. By using such a setting, differences in the basal activity of metabolically active tissues will become visible without confounding effects of, for example, differences in insulation or heat loss. There are several reports showing similar (17,20,95) or even higher (27,35) levels of oxygen consumption per whole animal in *ob/ob* mice at thermoneutrality than in control mice.

It thus appears that basal metabolic activity of *ob/ob* mice under thermoneutral conditions is essentially the same as in their lean controls. *Ob/ob* mice are thus not hypometabolic and their obesity seems to be entirely a result of their excessive food intake.

## Does BAT Atrophy Cause the Cold Intolerance and (Apparent) Decreased Energy Expenditure?

As outlined previously, both the development of massive obesity in *ob/ob* mice, as well as their acute cold intolerance, have been discussed as being partially from defects in energy metabolism. It appears, however, that there is little evidence to support a role for reduced energy expenditure in the development of their obesity. While it may seem unmotivated in a retrospective view, considerable efforts have been made to find the underlying cause of the apparent low metabolism of *ob/ob* mice, and even if the hypothesis of reduced metabolism in obesity appears not to be sustainable, the defect underlying the acute cold intolerance of *ob/ob* mice is still not fully understood. We therefore review here the existing literature on BAT nonshivering thermogenesis in *ob/ob* mice and its potential implications for their cold intolerance.

Initially, defects in Na+/K+-ATPase in skeletal muscle, kidney and liver had been discussed to contribute to the apparent hypometabolism of *ob/ob* mice (96–99). However, findings by Himms-Hagen (100) and Trayhurn (20,95) drew attention to an entirely different organ, BAT (26).

**Figure 3.**
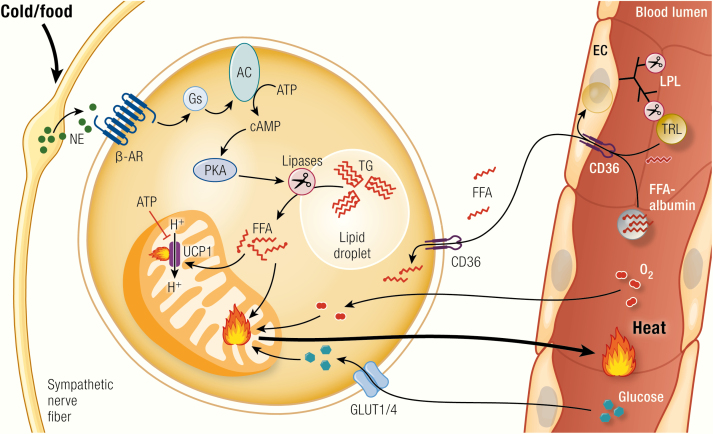
Brown adipocyte function. Upon activation of the sympathetic nervous system following cold exposure or dietary stimuli, norepinephrine (NE) is secreted from nerve endings and binds to β-adrenoceptors (β-AR) at the plasma membrane of brown adipocytes. This triggers G_s_-protein (G_s_)-coupled activation of adenylyl cyclase (AC), leading to the generation of cAMP, which will in turn activate protein kinase A (PKA). PKA activates lipolysis of triglycerides (TG), leading to the release of free fatty acids (FFA) that directly activate uncoupling protein 1 (UCP1) by overcoming the purine nucleotide (ATP, GTP, ADP, GDP) inhibition of this protein. The fatty acids may also fuel thermogenesis. Nutrients from the circulation are taken up from circulating triglyceride-rich lipoproteins (TRL; i.e., VLDL and chylomicrons) via the action of lipoprotein lipase (LPL) and the fatty acid translocase “cluster of differentiation 36” (CD36) on endothelial cells (EC), which may also internalize whole TRL particles. Also albumin-bound circulating free fatty acids can be taken up by brown adipose tissue. Another important source of fuel and precursors for *de novo* lipogenesis is circulating glucose, which is taken up via pathways dependent on glucose transporter 1 and 4 (GLUT1/4). To nourish its exceptionally high oxidative capacity, BAT has also to take up substantial amounts of oxygen. The main product of BAT is heat, which is released and distributed in the body by the circulation.

BAT is the main site of adaptive nonshivering thermogenesis and together with shivering and muscle tone thermogenesis is therefore one of the main thermogenic organs in mammals. BAT, and under certain stimuli such as prolonged cold exposure also white adipose tissue (WAT), namely brown-like adipocytes residing in WAT, called brite (101) or beige (102) adipocytes, are the only sites of expression of the mitochondrial uncoupling protein 1 (UCP1). UCP1 is the only protein mediating adaptive nonshivering thermogenesis (103,104). It does this by uncoupling the electron transport chain from ATP synthesis by facilitating proton transport over the inner mitochondrial membrane, thereby releasing energy as heat. Cold exposure leads to release of norepinephrine (NE) at sympathetic nerve terminals in BAT, activating adrenoceptors on the brown adipocytes, and, via adenylyl cyclase, leading to the formation of cAMP and to protein kinase A activation (Fig. 3). This will in turn activate lipolysis, thereby fuelling and supposedly activating thermogenesis (103). Activated BAT will subsequently need to take up large amounts of fuel, namely triglycerides (105–107), fatty acids (106,108–110), and glucose (106,111–113), as well as a substantial amount of oxygen (114,115) to support heat production (Fig. 3). Mice lacking functional UCP1 are acutely cold sensitive (116), but gradual exposure to cold can overcome their cold intolerance (104,117,118), probably through training effects on shivering endurance.

Being a main source of extra metabolism, defects in BAT in *ob/ob* mice seemed a likely candidate site of the apparently reduced metabolism in the *ob/ob* mice. Indeed, a plethora of studies have stated that there are some signs of defects in BAT in *ob/ob* mice. The effects of the *ob* mutation on different parameters of BAT function will be discussed separately in the following sections. In general, the evidence speaks against an atrophy of BAT in the *ob/ob* mice.

## Cold Tolerance Experiments Reflect Shivering – and not Nonshivering – Thermogenesis

An animal housed at a thermoneutral or subthermoneutral temperature will possess an amount of BAT adequate for its needs at that temperature; it will not possess a reserve capacity. The “reserve” that the animal has for a sudden extra cold challenge is to decrease heat loss, and to shiver. Thus, an acute cold tolerance test mainly measures the ability of the animal to initiate sufficient shivering thermogenesis (and to reduce heat loss). An increased capacity for adaptive nonshivering thermogenesis will only be recruited gradually with increasing time of cold acclimation (weeks) and can thus not be assessed by simply measuring the body temperature of mice during an acute cold challenge (hours) (67,119). The increase in energy expenditure upon an acute cold challenge mainly reflects the energy costs (and thus heat production) of shivering thermogenesis. To explain the cold sensitivity observed in such acute cold exposure experiments by a lowered nonshivering thermogenesis capacity is therefore only rarely relevant, but much experimental effort has been directed to examine this.

## Measuring Body Temperature Does Not Inform about BAT Thermogenesis - nor Does Measuring only BAT Temperature

During the “cold tolerance tests” described above, measurement of core body temperature is usually used to evaluate the thermogenic capacity of the animal. Also, for measurement of basal thermogenic capacity at a given temperature, core body temperature measurements are widely used. However, this type of analysis does not yield any direct information about BAT-derived thermogenesis. The core body temperature of an animal is tightly controlled, and a deficiency in the capacity of one effector (such as BAT) will be compensated by the initiation of other heat-producing and heat-conserving mechanisms.

Similarly, measurement of BAT temperature using infrared cameras or implantable devices does not necessarily reflect BAT activation. This is because a higher temperature of BAT can also be caused by a higher core body temperature, resulting from metabolism at other sites. Thus, when measuring BAT function, BAT temperature must always be related to core temperature (and should be warmer than the core, if the BAT is active). Despite the inherent technical limitations of such analysis, it may be noted that there are studies in *ob/ob* mice measuring BAT temperature (51) or “BAT minus core” temperature (19) after adrenergic stimulation of BAT. In both cases, BAT function appeared to be unaltered in *ob/ob* mice.

## Is BAT Recruitment Impaired?

**Figure 4.**
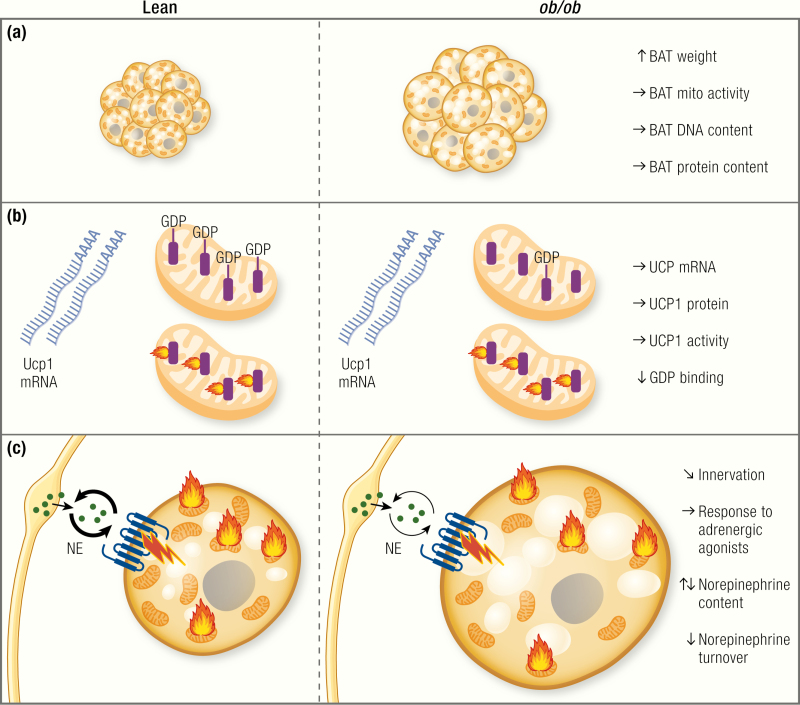
Is BAT function affected in *ob/ob* mice? An apparent defect in brown adipose tissue (BAT) recruitment has repeatedly been discussed to be the cause of the apparent cold intolerance and low energy expenditure. (A) However, despite higher tissue weight, which is mainly due to lipid accumulation, total protein content and DNA content, as well as cytochrome *c* oxidase activity are unchanged or even higher in *ob/ob* mice. Blue circles, nuclei; brown structures, mitochondria; yellow circles, lipid droplets. Note that per field/volume unit, the tissue looks atrophied, but the total amount of mitochondria is the same. (B) Uncoupling protein 1 (UCP1) is the only protein capable of performing nonshivering adaptive heat production. Although its mRNA levels normalized to housekeeping gene levels tell little about activity, the levels are a marker of acute sympathetic activation of the tissue, and, in steady-state situation, total tissue UCP1 mRNA levels correlate with the thermogenic capacity. Most reports show unaltered levels of UCP1 mRNA in *ob/ob* mice, whereas others show slightly reduced levels. Total UCP1 protein content of the tissue, which corresponds to the total thermogenic capacity, is largely unaltered. Purine nucleotides bind to UCP1, thereby inhibiting its activity. GDP binding can be used as a measure of UCP1 content and activation status. Surprisingly, despite unaltered protein levels of UCP1, GDP binding is generally found to be lower in *ob/ob* mice. This may correspond to lower sympathetic activation, but this is still not understood. (C) The activity of brown adipose tissue is regulated mainly via the sympathetic nervous system. Some reports show altered innervation patterns of BAT in *ob/ob* mice. Data on norepinephrine content of the tissue are inconclusive. Most reports state lower norepinephrine turnover in BAT of *ob/ob* mice. The response to pharmacological adrenergic stimulation is largely unaltered in *ob/*ob mice on the C57BL/6 background, but it is reduced on the Aston background.

The most obvious and visible phenotype of *ob/ob* BAT is the massive increase in the wet weight of the BAT depot (18,35,41,42,73,95,100,120–131) (Fig. 4A). Depending on the strain and treatment, *ob/ob* mice display BAT weights of up to 2 to 3 g (18,42), whereas in lean mice, the weight is usually in the range of about 100 mg. However, BAT weight cannot be seen as a marker of tissue activity. The weight of BAT increases upon cold acclimation, and this is paralleled by an increase in activity, but BAT weight also increases under conditions of inactivity, because of accumulation of lipids. Thus, simply measuring BAT wet weight does not reveal information about its activity (26,67,119,132). Indeed, as judged by histology (35), measurement of lipid content (133) or visual inspection (e.g., (73,100)), the lipid content of BAT is massively increased in *ob/ob* mice. This accumulation of lipids has been interpreted as reflecting an atrophy of the tissue (i.e., massive lipid accumulation from metabolic inactivity), but it may merely reflect a general “filling” of all lipid depots.

A more informative way of measuring the state of BAT recruitment is the assessment of BAT *total* protein content because higher activity will also be associated with higher protein content, irrespective of the lipid content and wet weight of the tissue. Several studies have measured the total protein content of BAT of *ob/ob* mice. Despite the apparent atrophy of the tissue, most studies actually found the total protein content of BAT to be similar in lean and *ob/ob* mice (35,100,126,127,131,134,135) or even higher in *ob/ob* mice under standard conditions (120,121,136) or after cold acclimation (126,131) (Fig. 4A). Also, the DNA content of the tissue is similar in lean and *ob/ob* mice, indicating similar (131,135) or even higher (18) cell numbers in *ob/ob* (Fig. 4A). Based on these findings, a general atrophy of BAT does not seem to exist in *ob/ob* mice (Fig. 4A).

## Do *ob/ob* Mice Have Defective BAT Mitochondria?

Because nonshivering heat production is located to the mitochondria, general disturbances in BAT mitochondria have been discussed as being the cause of the acute cold intolerance and the apparently low energy expenditure in *ob/ob* mice. However, the total mitochondrial yield and mitochondrial protein content are similar in lean and *ob/ob* mice (100,121) (Fig. 4A).

Although the total amount of mitochondria thus seems to be unchanged, altered ultrastructure could change the activity of the BAT mitochondria in *ob/ob* mice. Several articles report unusual ultrastructure of mitochondria isolated from BAT of *ob/ob* mice (36,120,121), an effect that can be normalized by cold exposure (121). The nature of this change in ultrastructure is not understood, but it seems to be corrected by tissue activation and thus most likely reflects metabolic inactivity of *ob/ob* BAT under the conditions used in these studies.

Several studies assessed mitochondrial activity by measuring cytochrome *c* oxidase activity. Most reports, however, found the total cytochrome *c* oxidase activity in BAT of *ob/ob* mice to be unaltered (100,135) or even increased (18,120,121,131) (Fig. 4A). Likewise, similar total levels of mitochondrial β-oxidation activity have been reported in *ob/ob* mice and WT mice at room temperature and after cold exposure (134).

Some authors report lower cytochrome *c* oxidase activity per milligram of protein in *ob/ob* BAT (120,133), lower mitochondrial β-oxidation activity per milligram of protein (134) or lower total succinate dehydrogenase activity (122). However, for some of these studies (120,133), the differences probably arise from different ways of expressing the activity data. As seen in (120), the enzyme activity per milligram of protein may well be reduced. However, the *ob/ob* mice have higher total BAT protein content, thus leading to equal amounts of *total* cytochrome *c* oxidase activity. As an estimate of the total mitochondrial content and oxidative capacity of the BAT depot, and from the animal’s point of view, this is the relevant parameter. The overall interpretation is thus that there is no general defect in oxidative capacity in BAT of *ob/ob* mice (Fig. 4A). Thus, if the apparent cold intolerance of the *ob/ob* mice is really based on defects in BAT, it should be a specific effect on UCP1-mediated proton conductance.

## Do *ob/ob* Mice Have Reduced Levels of UCP1?

In the late 1960s and early 1970s, it was clarified that heat production in BAT is located in the mitochondria and dependent on an uncoupling process mediated by a single protein (67,137). This protein was initially described as a polypeptide of 32,000 Da that was enriched in BAT mitochondria with increased abundance after cold exposure (138–140). The protein was isolated in 1980 (141) and called thermogenin, uncoupling protein, UCP, and later UCP1 (Fig. 3).

Even though UCP1 mRNA per housekeeping gene mRNA is not a readout of the thermogenic capacity of the tissue (in steady state, total UCP1 mRNA levels per BAT depot are however a good estimate of BAT capacity) (142,143), the change in UCP1 mRNA can be used as a measure of the acute adrenergic activation of the tissue. Several groups have measured UCP1 mRNA in BAT of *ob/ob* mice. Some found reduced levels of UCP1 mRNA in adult *ob/ob* mice at thermoneutrality (42), room temperature (73,144), or after cold exposure (42). In contrast, others found similar (53) or only slightly reduced levels that increased equally in lean and *ob/ob* after treatment with the β _3_-adrenergic agent BRL26830A (145). UCP1 mRNA at 28°C was the same in *ob/ob* and lean mice, and *ob/ob* mice responded normally to cold with an increase in UCP1 mRNA (43) (Fig. 4B).

However, UCP1 mRNA per housekeeping gene mRNA does not correspond to the total thermogenic capacity of the tissue (there is, for example, a dramatic increase in UCP1 mRNA within the first hours of cold exposure that does not translate into higher protein levels at this point (103,142,146)). Thus, particularly during a transition period, levels of UCP1 *protein* are much more relevant for estimation of the thermogenic capacity of the tissue (142,143). When measuring UCP1 protein expression, most reports on *ob/ob* mice state similar levels of UCP1 protein, first indicated by the observation that the levels of polypeptides of ~32,000 Da are similar in lean and *ob/ob* (120,121,134).

As reviewed in (142,147), and exemplified in (35), it is the *total* amount of UCP1 protein per BAT depot, and not the UCP1 concentration in the tissue (per milligram of protein, as seen on a Western blot) that determines the total thermogenic capacity of the animal. Indeed, and in line with the data on mitochondrial content, the total levels of UCP1 protein per BAT depot in *ob/ob* mice were equal to those in lean mice at thermoneutrality (41), at room temperature (35) or after cold acclimation (148) (but note a delayed cold response in UCP1 protein induction (41,148)).

There are some reports showing a reduced concentration of UCP1 on Western blots (144). However, this technique reflects UCP1 levels per mg protein. Because total protein content of BAT in *ob/ob* mice is increased (120,121,136), it is reasonable to assume that total UCP1 levels also in these mice were similarly elevated to those in the lean controls.

The previously mentioned results show, despite some variability, that there does not seem to be an inherent reduction in UCP1 total protein in BAT of *ob/ob* mice (Fig. 4B). However, despite equal UCP1 levels, BAT thermogenic capacity may still be lower in *ob/ob* mice. A valuable tool for assessment of UCP1 capacity *in vivo* is the measurement of the response to norepinephrine (NE).

## Do *ob/ob* Mice Have Reduced Potential Capacity of UCP1?

A measure of UCP1 activity *in situ* is the increase in metabolism seen in the intact mouse in response to the injection of the adrenergic agonist NE or of more selective β _3_-adrenergic agonists such as BRL26830A or CL316,243. The increase in oxygen consumption following the injection into conscious (for CL316,243 experiments) or pentobarbital-anesthetized (for NE experiments) animals correlates principally with the total amount of UCP1 (67,119). In the case of NE, this occurs on top of a UCP1-independent, nonrecruitable/nonadaptive adrenergic thermogenesis, a pharmacological response in many tissues following a high bolus dose of sympathetic transmitter.

Some studies found reduced levels of oxygen uptake or oxygen consumption following injection of NE in anaesthetized *ob/ob* mice (120,121). In contrast, other studies found similar increases in oxygen consumption in *ob/ob* and lean mice following NE injection (19,35) or even higher responses in oxygen consumption (70,76). Also, the oxygen consumption response to other compounds such as BRL26830A and CL316,243 (19) was similar in lean and *ob/ob* mice. The nature of these discrepancies in adrenergic response is presently unclear. Although most reports do not seem to support the idea of lowered thermogenic capacity of BAT in *ob/ob* mice, there is thus still the possibility of a somewhat delayed or disturbed response to adrenergic stimulation in *ob/ob* mice.

## Is Nonshivering Thermogenesis Capacity Reduced in *ob/ob* Mice—GDP Binding?

UCP1 is activated by fatty acids to overcome the innate inhibition of UCP1 caused by cytosolic ATP and other purine nucleotides (67,103). (For the current debate about the role of intracellular lipolysis for BAT activity, see (149–151)). The number of nucleotide (GDP/ATP) binding sites should thus be proportional to the amount of UCP1 in the tissue. Nonetheless, there is a still unexplained “unmasking” phenomenon (103,152,153). In this phenomenon, the number of binding sites for GDP is increased (often doubled) by, for example, cold exposure within a time frame that would not allow for changes in UCP1 protein levels. Even in mice treated with cycloheximide to inhibit protein synthesis, this rapid increase in GDP-binding is observable (154). Thus, the results of [^3^H]GDP-binding assays can be difficult to interpret as to whether the binding reflects activity of the mitochondria or the amount of UCP1. Because [^3^H]GDP-binding was initially used extensively as a readout of BAT activity in *ob/ob* mice, the results will be discussed here.

The data on GDP-binding in mitochondria from *ob/ob* mice are, in contrast to most other data discussed here, surprisingly homogeneous. The binding of GDP to BAT mitochondria in an inactive state (i.e., in mitochondria isolated from mice housed at thermoneutrality) or at room temperature has repeatedly been reported to be lower in *ob/ob* mice (41,44,100,120,121,125–127,129,134,135,155), and only a few articles reported unchanged basal levels (18,19,148) (Fig. 4B).

The acute activation of BAT usually results in an “unmasking” of the GDP-binding sites in isolated mitochondria, which may be related to conformational changes in the UCP1 protein (103) or perhaps to mitochondrial swelling (156,157). The increase in binding sites upon acute cold activation of the tissue can thus be interpreted as a measure of the activation of nonshivering thermogenesis. Several groups found indications of attenuated responses to cold of GDP-unmasking in *ob/ob* BAT. Acute cold exposure did not increase GDP-binding in *ob/ob* mice, whereas lean mice showed the expected increase (41,100,120,121,126), or the increase in GDP-binding sites was delayed by several days (148). One report, however, stated higher GDP-binding in *ob/ob* BAT mitochondria than in lean controls after treatment with norepinephrine or the β _3_-adrenoceptor agonist BRL26830 (19).

Some reports have shown that GDP-binding in BAT mitochondria of *ob/ob* mice is normalized after cold acclimation for several weeks (126,134) and increased levels after feeding calorie-rich diets (18,125,127), after adrenalectomy (128), or after T4 treatment (120), which implies increased UCP1 protein synthesis from these treatments. These data have been interpreted as reflecting initially reduced levels of UCP1 in BAT of *ob/ob* mice. However, as outlined previously, based on immunoblotting studies, this does not seem to be the case. The reduced basal levels of GDP binding found in some studies are thus still unexplained, especially since Milner and Trayhurn (148) showed equal amounts of GDP per UCP1 in *ob/ob* and lean mice under thermoneutral conditions. The diminished or delayed “unmasking” response may be a manifestation of a reduced activation ability of the tissue upon acute cold challenge, which could result from improper sympathetic innervation, reduced NE secretion or disturbances in the intracellular adrenergic signalling cascade in brown adipocytes of *ob/ob* mice (Fig. 4BC).

## Is There a Defect in Sympathetic Stimulation of BAT in *ob/ob* Mice?

The slower activation response of BAT in *ob/ob* mice in response to acute cold exposure may reflect defects in the sympathetic innervation of the tissue. The activity of the sympathetic nervous system can be estimated by measuring NE content and turnover within the tissue.

Several studies reported slightly lower *total* NE content in BAT of *ob/ob* (123,130,131) that normalized with cold exposure (130,131), whereas others showed a higher content at several temperatures (124) (Fig. 4C).

Another readout of BAT sympathetic activation is the turnover of NE. Higher turnover reflects higher activity of the tissue. Several studies reported lower NE turnover in BAT of *ob/ob* mice under thermoneutral conditions, at room temperature (123,130,131,158), or in response to cold exposure (131) or acute exposure to 26°C (124) (Fig. 4C). However, some found the turnover rates to be similar at thermoneutrality (18,131), which may be of more relevance, because it reflects the basal state of the tissue, where no confounding effects of cold-induced activation are visible.

Despite data showing similar NE-turnover rates in BAT of *ob/ob* mice, it seems that there is some delay in NE secretion in response to cold, which is in line with the slower increase in GDP-binding sites (Fig. 4BC). To investigate this, Seydoux et al. (133) performed electrical and adrenergic stimulation of BAT *ex vivo* and found a reduced response in NADPH-fluorescence in BAT from *ob/ob* mice following stimulation. This difference was reduced when mice were cold-acclimated. Begin-Heick et al. (122) found equal basal activity of adenylyl cyclase in BAT of *ob/ob*, but a reduced response to adrenergic stimulation.

Thus, there seems to be some defect in the responsiveness to physiological adrenergic stimulation of the tissue (at least in some reports). These differences could partially arise from differences in innervation, since it can at least partially be overcome by pharmacological stimulation *in vivo.* Staining for catecholamines of BAT of lean and *ob/ob* mice have shown innervation mainly around blood vessels in *ob/ob* mice, while lean mice showed dual innervation on vessels and adipocytes (159). These represent 2 different populations of nerves, the vessel-innervating nerves being NPY- and tyrosine hydroxylase-positive, whereas the adipocyte-innervating nerves are only positive for tyrosine hydroxylase (i.e., norepinephrine containing), and presumably other neuropeptides (160). It thus seems possible that the adipocyte-innervating subpopulation should require leptin signalling for its development or existence. As a result, fewer contact sites between nerves and adipocytes have been observed in *ob/ob* BAT at room temperature (159). In accordance with these findings, it has been reported that leptin can directly stimulate axon outgrowth of sympathetic neurons *in vitro* (161), an effect that is therefore presumably impaired in the *ob/ob* mice.

However, as outlined previously, the delayed response in BAT activation observed in some studies does not necessarily explain the acute cold intolerance of *ob/ob* mice, because the response to acute cold relies mainly on shivering thermogenesis, whereas nonshivering thermogenesis in BAT is only recruited gradually during the course of cold acclimation (103). It may, however, reflect a general tendency toward lower sympathetic activation in these mice, a state that could also result in a lower degree of sympathetically stimulated lipolysis in WAT, which could limit substrate availability for shivering thermogenesis.

The initiation and maintenance of shivering thermogenesis requires proper fiber type in the muscle tissue, as well as sufficient fuel supply to muscles. *ob/ob* mice on a Balb/c background are less susceptible to cold-induced acute hypothermia than are *ob/ob* mice on the BL/6 background (23,24). Correspondingly, these Balb/c mice show higher rates of *ex vivo* lipolysis in white fat and higher activity-induced plasma glycerol levels, as compared with the less cold-tolerant C57BL/6 *ob/ob* mice (24). It may thus be speculated that a delayed or reduced initiation of lipolysis in *ob/ob* mice limits muscle fuel supply, thereby limiting the shivering response usually seen upon acute cold exposure. Indeed, some studies measuring lipolysis in *ob/ob* mice or in isolated *ob/ob* adipocytes found reduced levels of basal and of stimulated lipolysis (162,163), and leptin treatment has been shown to activate lipolysis via the sympathetic nervous system (164).

## BAT Acute Activation and Recruitment Seem Unrelated in C57BL/6 *ob/ob* Mice

Taken together, it can be concluded that BAT protein, DNA and mitochondrial content, mitochondrial activity, and UCP1 levels are unaltered in *ob/ob* mice under most conditions. The *ob/ob* mice, at least in most reports of *ob/ob* on the C57BL/6 background, respond normally to pharmacological stimulation of BAT thermogenesis. The metabolic response to sympathetic activation during acute cold exposure, however, seems to be delayed, as judged by GDP binding, NE turnover, or the rate of increase in UCP1 protein in the cold. The basal activation of the tissue seems to be unaltered, and long-term cold acclimation can overcome the defect and lead to normal levels of activity. It may be speculated that the observed differences in innervation can partially explain this phenotype. How this relates to the function of leptin is unclear.

The discrepancy between unchanged UCP1 levels and altered sympathetic innervation is not readily understood. In the general concept, in which all UCP1 expression in BAT relies on proper sympathetic signalling, reduced sympathetic input should necessarily result in lower UCP1 levels. This is clearly not the case in *ob/ob* mice. It may be speculated that the (reduced) adrenergic stimulation of the tissue, while not being sufficient to mediate full activation of the tissue, may nevertheless be high enough to induce and maintain basal UCP1 expression. In accordance with this, both denervated BAT (165–170), as well as BAT from animals with defective NE production (171), or β-adrenoceptor deficiency (172), still displays basal UCP1 expression. Several hormonal factors have been discussed to mediate effects of UCP1 expression and BAT thermogenesis independently of sympathetic tone (173,174). It seems thus possible that one or several of these factors, which include hormones such as secretin (175), FGF21 (176), or natriuretic peptides (177), as well as metabolites such as bile acids (178,179) or signaling lipids (180), mediate the basal UCP1 expression in *ob/ob* mice.

## No General Defect in Energy Metabolism in C57BL/6 *ob/ob* Mice

All the findings presented so far indicate that although *ob/ob* mice clearly can become hypothermic when acutely exposed to cold temperatures, they are able to defend their body temperature at subthermoneutral temperatures given they have had enough time to acclimate. However, they defend a lower body temperature at subthermoneutrality. *ob/ob* mice are clearly not hypometabolic but rather hypermetabolic and are able to respond normally to cold exposure in increasing their metabolism. Decreased metabolism thus does not contribute to the development of obesity in these mice.

## The Aston *ob/ob* Strain Does Display BAT Defects

As mentioned, the *ob* mutation has been maintained on several genetic backgrounds. Although the most commonly used strain is the *ob/ob* on the C57BL/6 background from the Jackson Laboratories, a substantial number of classical studies on thermoregulation have been performed in the so-called Aston strain.

**Figure 5.**
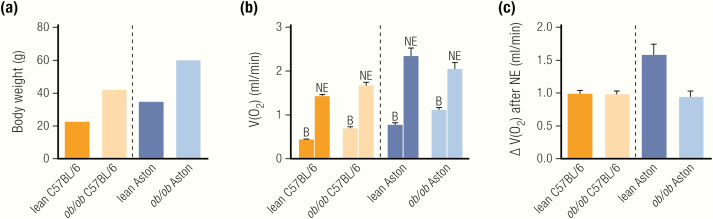
The difference in response to adrenergic stimulation in C57BL/6 and Aston *ob/ob.* Values are taken from (19). (A) Body weight of *ob/ob* is higher in both strains, with the Aston mice generally being larger, similarly to most other outbred strains. (B) Metabolism per whole mouse in pentobarbital-anesthetized lean and *ob/ob* mice of the C57BL/6 and the Aston strain are shown before and after injection of norepinephrine. Basal metabolism is higher in *ob/ob* mice of each strain, compared with lean controls. Aston mice display higher basal metabolism than C57BL/6 mice, most likely a reflection of their higher body weight (A). Norepinephrine causes responses in all groups, with Aston mice displaying a higher response. B, basal metabolism; NE, norepinephrine-induced metabolism. (C) Quantification of the norepinephrine-induced increase over baseline shows reduced responses in *ob/ob* mice of the Aston strain as compared with their lean controls, but no difference between lean and *ob/ob* on the C57BL/6 background.

To increase litter size and growth rate, *ob/ob* mice from the Jackson Laboratories were crossed into 2 local outbred mouse strains at Edinburgh University in 1957. These were then further crossed into local non-inbred strains and in 1966 transferred to Aston University, Birmingham (181,182). These mice therefore carry the same point mutation in the leptin gene as those on the C57BL/6 background (182) but display a somewhat more dramatic phenotype. This may be partially because outbred mice are generally larger than inbred mice (see Fig. 5A). As outlined previously, on the outbred Aston background, *ob/ob* can reach body weights of 70 g (20) to 90 to 100 g (38,70), whereas the *ob* mutation on the inbred C57BL/6 background only leads to body weights between 40 g (17) and around 60 g (18) (Fig. 5A).

Similar to the BL/6 *ob/ob*, the Aston *ob/ob* mice are severely cold intolerant, and the acute cold intolerance of *ob/ob* has even been suggested to be a useful tool to “genotype” the offspring of heterozygous +/*ob* matings. Indeed, Trayhurn et al. (44) and Thurlby and Trayhurn (45,183) were able to predict the development of obesity in offspring of such heterozygous matings using a cold-exposure test in young 12-day-old mice, an age at which there is no obvious difference in body weight between wild-types and mutants.

Although most studies using C57BL/6 *ob/ob* did not find signs of reduced energy expenditure per whole mouse, the Aston *ob/ob* mice seem to be hypometabolic at subthermoneutral temperatures (20). These mice also become severely obese even when pair-fed to normophagic lean littermates (26,91), just as the C57BL/6 *ob/ob*. However, as discussed, such analyses are confounded by the initiation of torpor (91). When energy expenditure in the Aston mice was calculated based on intake and excretion of energy, even Aston *ob/ob* were not hypo- but rather hypermetabolic (127).

BAT recruitment and adrenergic responsiveness seem to be clearly different in *ob/ob* mice on the different backgrounds. The Aston mice display lower *total* cytochrome *c* activity under thermoneutral and acute cold conditions (148), while cold acclimation led to higher levels in *ob/ob* than in lean controls (148).

Levels of UCP1 protein measured by radioimmunoassay are lower in older, but not in young, Aston *ob/ob* (129). Even lower *total* levels of UCP1 under thermoneutral conditions have been reported (148). This is in line with reports showing lower GDP-binding in these mice (44,125,127,129,135), although the physiological significance of this remains unclear.

In addition, also the thermogenic response to NE injection was lower in Aston *ob/ob* mice (20), and Thurlby and Trayhurn (95) found reduced total blood flow into BAT of Aston *ob/ob* following norepinephrine injection, compared with lean controls. Of note, blood flow under basal conditions was even higher in *ob/ob* mice (95).

Wilson and colleagues (19) directly compared the adrenergic responsiveness of *ob/ob* mice from both strains. They reported that *ob/ob* on the C57BL/6 background respond normally to adrenergic stimulation, whereas Aston *ob/ob* mice show a 50% reduced response (see Fig. 5B, C). Taken together, there is compelling evidence that the degree of recruitment of BAT in the Aston *ob/ob* mice is indeed lower than in the lean mice (whereas this is probably not the case in C57BL/6). The nature of this strain difference is presently unclear. It may be said that the Aston *ob/ob* become even more obese than C57BL/6 *ob/ob* mice, but they are also larger under wild-type conditions, as are most outbred strains (see Fig. 5A). Thus, the relative increase in obesity caused by the *ob* mutation appears to be similar in BL/6 and Aston *ob/ob* mice compared with lean controls, and there is no evidence that the reduced BAT activation actually promotes obesity development.

## Effects of Loss of Leptin Receptors: *db/db* Mice

In 1959, a new mutation was discovered at the Institute of Animal Genetics in Edinburgh, that led to a similar obese phenotype as the *ob* mutation (184). This mutation was then called the “adipose” mutation (6,184). A few years later, in 1966, another mutation, leading to massive obesity, was identified in a colony of C57BL/Ks mice at Jackson Laboratories, the so-called “diabetic” (*db/db*) phenotype (6,9,185,186). Adipose and diabetic were later both found to be located in a gene on chromosome 4 (6) and it was later established that the *db/db* mouse lacked the functional hypothalamic leptin receptor LepRb. A G→T mutation in the gene caused abnormal splicing and led to a 106 nucleotide insertion in the transcript, resulting in premature termination of the signaling receptor isoform LepRb and loss of its signal-transducing abilities (187,188).

When expressed on a C57BL/6 background, the development of obesity, insulin resistance, and lack of fertility are similar in *ob/ob* and *db/db* mice (189). When expressed on a C57BL/Ks background, which is the background mainly used in studies on thermoregulation in *db/db* mice, the phenotype of the *db* mutation, at least concerning glucose metabolism, is much more dramatic than on BL/6 (189). Similarly, *ob/ob* mice on the C57BL/Ks background show a more dramatic phenotype with respect to glucose metabolism than *ob/ob* on the C57BL/6 background (10). Thus, a direct comparison between results obtained in *db/db* mice and in *ob/ob* mice might be hampered by these background strain differences.

Similarly to *ob/ob* mice, the *db/db* mice become obese even when pair-fed to lean mice (190,191) but again, this may be related to the torpor that is most likely induced by food restriction, although this has not been directly shown. The *db/db* mice also show lower body temperature at subthermoneutral temperatures (47,192). When acutely exposed to the cold, the *db/db* mice also become severely hypothermic (47,192), while displaying stable, but lower, body temperatures and functional diurnal rhythmicity of body temperature when gradually exposed to the cold, or when maintained under conditions of mild cold exposure (23°C) (192), indicative of a reduction in thermoregulatory effector thresholds similar to the situation observed in *ob/ob* mice.

Levels of basal energy expenditure per mouse under thermoneutral conditions were similar in lean and *db/db* mice (192). They may, however, display a somewhat reduced increase in energy expenditure in response to cold exposure (192).

The basal levels of UCP1 mRNA are similar in lean and *db/db* mice (43), and the induction of UCP1 mRNA in response to cold was only slightly reduced in *db/db* (43), indicative of functional adrenergic responsiveness. In line with data obtained in Aston *ob/ob*, but in contrast to most data from C57BL/6 *ob/ob*, GDP-binding in *db/db* mice at 23°C is reduced, as is tissue protein and DNA content and cytochrome *c* oxidase activity (193). Injection of norepinephrine caused a reduced response in *db/db* mice acclimated to 23°C (192), indicative of a reduced BAT capacity.

These data seem to imply that there is some degree of defect in BAT in these animals, in line with the data obtained in Aston *ob/ob* mice, but in contrast to the results obtained in most studies using C57BL/6 *ob/ob* mice. Although there is no comparative study on thermoregulatory differences between C57BL/6 and C57BL/Ks mice, the differences observed in *ob/ob* and *db/db* mice on either background with respect to insulin resistance, glycemia, and pancreatic islet integrity at least open for the possibility of differences in thermoregulation between these strains related to glucose metabolism.

## Effects of Loss of Leptin Receptors: *fa/fa* Rats

Rats with genetic obesity have also been identified, namely the so-called “fatty” (*fa/fa*) or “Zucker” rat (194). This mutation occurred in the so-called 13M rat stock and is characterized by massive obesity, infertility, and elevated plasma lipids, as well as hyperinsulinemia (6,194). The mutation causing the phenotype is in the same gene as the *db* mutation (195). The mutation is a single nucleotide substitution from A to C, leading to a Glu to Pro substitution at position 269 of the protein (196). This is within the C domain of the receptor, a domain common to all LepR isoforms. The long, signaling isoform of the receptor is still able to bind leptin in *fa/fa* rats, but not to induce signaling events (196).

*Fa/fa* rats, just as *ob/ob* and *db/db* mice, are characterized by hyperphagia, which has been discussed to partially, but not fully, explain their phenotype, because animals pair-fed to lean animals still gained more weight than lean controls (6,197–199). Although rats are not known to undergo torpor, there may be confounding effects of caloric restriction, as in the *ob/ob* mice. Similarly to *ob/ob* mice, reduced metabolism following restricted feeding is also observed in *fa/fa* rats (198), while levels of energy expenditure in *ad libitum-*fed lean and *fa/fa* rats of 48 to 84 days of age are similar (198). Only when expressed per gram of body weight, which was doubled, did the fatty rats appear hypometabolic. This was seen at both 22°C and 28°C. Also, a study (200) reported 30% lower oxygen consumption per gram body weight, whereas body weight was 50% higher; the reduced metabolism is thus again a normalization artefact. The same seems to be true for the adult rats in another study (201), where oxygen consumption was also normalized to body weight (but note also slightly reduced levels in young preobese rats). Indeed, normalization to body protein, representing metabolically active tissue mass, revealed no difference in the metabolism of lean and *fa/fa* rats (202). Levels of resting energy expenditure under thermoneutral conditions are also similar in lean and *fa/fa* rats (203) (but note the lower energy expenditure in this study when calculated based on intake and excretion). Thus, *fa/fa* rats are also not obviously hypometabolic.

Similarly to the *ob/ob* and *db/db* mice, young *fa/fa* rats display lower body temperature at subthermoneutral temperatures (204,205); the same is seen in adult rats after exposure to the cold (206) but not at room temperature (207) that may be closer to thermoneutrality in rats. Accordingly, similarly to the case for *ob/ob* and *db/db* mice, defects in BAT have been discussed to be causative of the lower body temperature – even though body temperature is not a readout for BAT function.

BAT weight is significantly higher in *fa/fa* rats (208), whereas measurement of parameters of BAT recruitment in *fa/fa* rats housed at ≈27°C revealed similar protein content (203,209) and succinate cytochrome *c* oxidoreductase activity (209). Direct measurement of UCP1 content per mg protein showed similar levels of UCP1 in young lean and *fa/fa* rats and lower levels in 12-week old *fa/fa* rats housed at 23°C (129). However, because total tissue protein content was not reported, the total thermogenic capacity cannot be estimated based on these values. GDP-binding in BAT mitochondria is reduced in *fa/fa* rats under thermoneutral (26°C) conditions (203,209). Others found the GDP-binding per milligram of protein also to be slightly lower in *fa/fa* rats following injection of NE (210) or in *fa/fa* rats of different ages, housed at 23°C (129). BAT total organ NE content was lower in *fa/fa* rats under basal levels and following cold exposure, whereas NE turnover was even higher in the cold (210).

The thermogenic response to NE has been reported to be lower in obese *fa/fa* rats acclimated to 22°C (208). However, this study reports the oxygen consumption per body weight, which was increased in the obese animals, and it is therefore unlikely that there is a true difference. They also report total blood flow per BAT under stimulated conditions to be slightly lower in *fa/fa* rats, while total BAT blood flow was even 4 times higher in *fa/fa* rats under basal conditions (208).

It thus seems that, although not displaying general disturbances in energy expenditure, just like *ob/ob* and *db/db* mice, the obese *fa/fa* rats show some signs of reduced BAT GDP-binding and NE content. Under thermoneutral conditions, however, BAT shows no signs of general atrophy. This is thus, despite some variability, in accordance with data obtained in the C57BL/6 or C57BL/Ks mouse models of leptin/leptin receptor deficiency (Table 1).

**Table 1.** Differences in Metabolism in Various Strains of Leptin-signaling Deficiency

Parameter	C57BL/6 *ob/ob*	Aston *ob/ob*	C57BL/Ks *db/db*	*Fa/fa*
Food intake	↑	↑	↑	↑
**B** **ody weight**	↑	↑	↑	↑
**Body temperature at thermoneutrality**	→	→	→	?
Body temperature below thermoneutrality	↓	↓	↓	↓
**Energy expenditure at thermoneutrality**	↑→	→	→	→
**Energy expenditure below thermoneutrality**	↑→	↓	↓	→
BAT weight	↑	↑	↑	↑
**BAT protein content**	→	↓	↓	→
UCP1 mRNA	→	?	→	?
UCP1 total protein	→	↓→	?	?
GDP binding	↑↓	↓	↓	↓
Sympathetic innervation and activity	↓	↓	↓	↓
NE response	→	↓	↘	↓→

The effects of leptin deficiency (*ob/ob*) on various metabolic parameters are shown in mice of the 2 most common genetic backgrounds (C57BL/6 and Aston), as well as the effects of leptin receptor deficiency in mice (C57BL/Ks *db/db*) and rats (*fa/fa*). Arrows indicate changes compared to the respective wild-type controls. Black arrows indicate similar findings in all 4 strains, whereas red arrows indicate parameters that are affected differently in the different strains. Question marks indicate parameters not measured in the respective strain. Arrows pointing in opposite directions indicate contradictory findings. Abbreviations: NE response, increase in energy expenditure in response to norepinephrine injection.

## The Effects of Leptin

In 1994, the Friedman laboratory determined the nature of the genetic defect in the *ob/ob* mice and thus identified leptin (16). Leptin (derived from the Greek word *leptos*, meaning “thin”) was the name given to the protein normally encoded by the *ob* gene. Leptin is a hormone secreted by adipocytes and that acts primarily in the hypothalamus (7). Leptin treatment leads to reduction in food intake and thus in body mass in *ob/ob* mice (52,211,212), demonstrating that it was indeed the lack of leptin that caused obesity in the *ob/ob* mice.

It was thus logical to also study the effects of leptin treatment and leptin replacement in *ob/ob* mice on body temperature regulation, energy expenditure, and BAT physiology. All studies on the effects of leptin treatment performed on *ob/ob* have been performed in mice on the C57BL/6 background. The results of these studies will be discussed in the following sections, but, as summarized previously, it should be remembered that the *ob/ob* mice do not really demonstrate any hypometabolic or BAT atrophy phenotype.

## Leptin is a Pyrexic Agent

**Figure 6.**
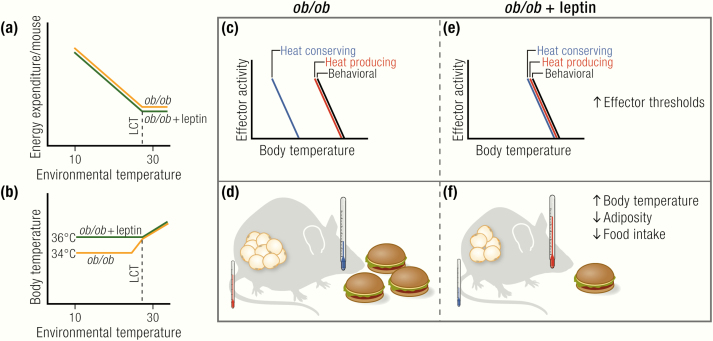
Leptin is a pyrexic and not a thermogenic agent. (A) Leptin treatment has no effect on energy expenditure in *ob/ob* mice under thermoneutral or subthermoneutral temperatures. (B) Leptin treatment acutely increases body temperature in *ob/ob* mice at subthermoneutral temperatures. (C, E) This effect is, however, not a thermogenic response, but a pyrexic (febrile) increase in body temperature because it leads to a seemingly higher “defended” body temperature through an increase in the activation threshold of heat-conserving, but not behavioral, effectors. The increase in body temperature following leptin treatment is not due to increased thermogenesis, but is mainly due to decreased heat loss. (D, F) Leptin replacement in *ob/ob* mice reduces food intake and adiposity. Additionally, it acutely increases body temperature in *ob/ob* mice, mainly by decreasing heat loss from the tail.

The leptin-deficient *ob/ob* mice display changes in the temperature threshold for their thermoregulatory effectors. They show a lower but defended body temperature at subthermoneutral temperatures. Therefore, leptin replacement in *ob/ob* mice is expected to normalize body temperature. Indeed, body temperature in *ob/ob* mice living at room temperature is increased after acute (within hours) (35), semiacute (within 2 days) (53) or chronic treatment with leptin (35,52,53,213,214) (Fig. 6B,D,F). In pair-fed animals, body temperature was only increased after long-term treatment (54). In some reports, there is also a slight positive effect of long-term leptin treatment on body temperature at thermoneutrality (35,214).

Thus, leptin clearly has an effect on body temperature in *ob/ob* mice (Fig. 6B,D,F). The slightly increased body temperature in *ob/ob* mice at thermoneutrality and the increase in body temperature back to wild-type levels below thermoneutrality point towards a pyrexic response (i.e., an increase in the defended body temperature) (Fig. 6C,E). The increase in body temperature, however, does not necessarily mean that thermogenesis is increased; also, alterations in heat retention can contribute to the pyrexic response (35,60).

## Leptin Is Not Directly Thermogenic in *ob/ob* Mice

Immediately after the discovery of leptin, it was reported that long-term leptin treatment of *ob/ob* mice led to increased levels of energy expenditure (52). However, as repeatedly discussed (35,65–67), the energy expenditure values reported in this article were “normalized” per body weight, and the mice lost a substantial amount of body weight (mainly inert fat mass (35)) during the course of the treatment. The values are thus only a reflection of a loss of body fat; when recalculated per animal, leptin had no effect on energy expenditure. Accordingly, several other groups reported energy expenditure to be unaffected by leptin treatment in *ob/ob* mice (35,213,215–217) or even decreased at 30°C, where a reduced thermic effect of feeding because of massive reductions in food intake is observable (35) (Fig. 6A). There is no acute effect of leptin injection on metabolic rate, neither in *ob/ob* mice nor in wild-type mice (35).

## Leptin Action on Body Temperature in *ob/ob* Mice Is Mainly Mediated by Changes in Heat Loss

The rapid increase in body temperature in *ob/ob* mice following leptin treatment in the absence of marked increases in energy expenditure implies that other thermoregulatory mechanisms than heat production must be used in this process. Behavioral thermoregulation is not affected by leptin treatment, as leptin-treated *ob/ob* mice, just like saline-treated control *ob/ob* mice, select temperatures close to 30°C when placed in a thermal gradient (35). Thus, heat loss must be reduced. Heat loss in the mouse is mainly achieved by vasoconstriction in the tail (218,219). Indeed, thermal conductance, a measure of heat loss, is markedly reduced following leptin treatment (35,213). This process is mainly mediated by the reduced tail heat loss (35) (Fig. 6DF).

## Does BAT Play any Role in the Response to Leptin in *ob/ob* Mice?

As concluded previously, *ob/ob* mice on a C57BL/6 background do not demonstrate an inherent defect in BAT as measured by tissue protein content, UCP1 mRNA and UCP1 protein, or the response to adrenergic stimulation. They do, however, just as *db/db* mice and *fa/fa* rats, display signs of a delayed response to cold exposure and reduced GDP binding. *ob/ob* mice on the Aston background do have reduced levels of UCP1 mRNA, UCP1 protein, GDP-binding, and sympathetic activation of BAT. Because some of the data, especially those obtained in Aston mice, implied BAT defects from leptin deficiency, the effects of leptin replacement on several parameters of BAT activation have been studied. However, this has not been performed in the more interesting target, the Aston mice, but only in the C57BL/6 model.

**Figure 7.**
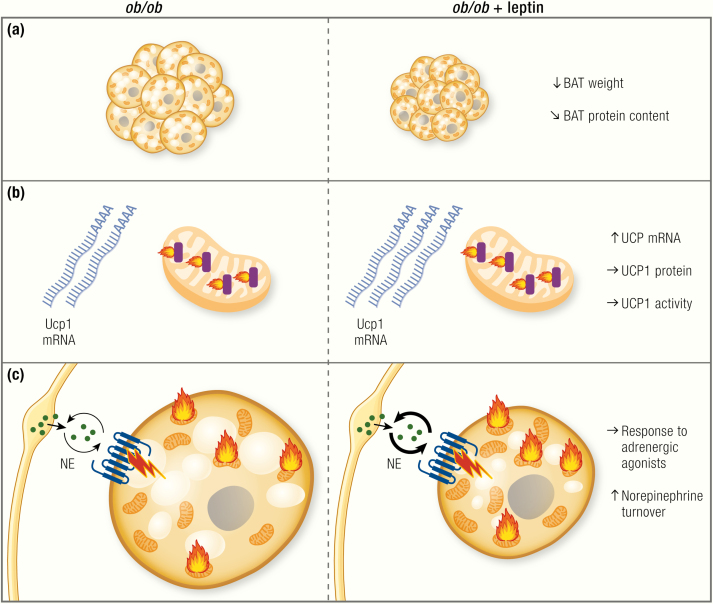
Does BAT play any role in the response to leptin? Data on the effects of leptin replacement on BAT function are less complete than the effects of leptin deficiency. (A) BAT weight is clearly reduced by leptin treatment and also total protein content is slightly decreased. Note that the tissue seems to go from an atrophied to an active state; however, this seems only to represent a transient lipolytic effect, as BAT is not recruited. (B) UCP1 mRNA has been reported to be increased following leptin treatment. This does not, however, translate into higher UCP1 total protein levels. (C) There are signs of increased sympathetic activity following leptin treatment, such as increased norepinephrine (NE) turnover. The response to adrenergic stimulation is, however, unaltered.

Leptin has been reported to slightly induce the levels of UCP1 mRNA in *ob/ob* mice (53) (Fig. 7B). Because of the short half-life of UCP1 mRNA (~4 hours (146,220–223)), this can be seen as a proxy for increased sympathetic activity and does not necessarily translate into higher UCP1 protein levels (142).

Indeed, lipolysis in BAT seems to be increased following leptin treatment, as judged by the reduction in tissue mass (35,53) and reduction in lipid droplet size in leptin-treated *ob/ob* mice (35) (Fig. 7A), which can, however, also be interpreted as being a simple reflection of the reduced food intake under these conditions. However, Collins et al. (224) also found increased NE turnover in BAT of *ob/ob* mice following leptin treatment (Fig. 7C), and leptin has been shown to increase lipolysis in a sympathetic nerve-dependent manner (164)

Some authors reported an increase in UCP1 protein levels on Western blot in BAT of *ob/ob* mice treated with leptin (35,144). However, as stated previously, it is the *total* UCP1 protein amount in the animal that best corresponds to the thermogenic capacity. Indeed, when calculated per whole animal, UCP1 levels in leptin-treated *ob/ob* mice are not different from the levels in untreated *ob/ob* mice, and also the NE response in the whole mouse is unaltered (35) (Fig. 7BC).

It thus seems that, although leptin does increase sympathetic tone, at least in leptin-deficient animals, leptin treatment does not necessarily result in increased levels of UCP1 or nonshivering thermogenesis (Fig. 7). This is congruent with the absence of effect of the *ob/ob* mutation on BAT thermogenic capacity, at least on the C57BL/6 background. Why the increased sympathetic activation would not translate into higher levels of UCP1 protein is currently unknown. Because *ob/ob* mice in general seem to be able to maintain high levels of UCP1 despite decreased sympathetic activity, they seem to display an enigmatic dissociation between sympathetic input and UCP1 levels, an observation clearly worthy of further investigation.

Since leptin treatment results in similar weight reduction in *ob/ob* mice and in *ob/ob* mice lacking UCP1 (217), it would appear that the weight-reducing effect of leptin can be fully explained by reductions in food intake.

## Leptin Has Some Pyrexic Effect in Wildtype Mice but Is Not Thermogenic

While the effect on body temperature seems to be rather clear in *ob/ob* mice, some effects were also observed in lean wild-type animals that *per se* should not lack leptin signaling and are not hypothermic. Acute leptin treatment in wild-type mice (35), as well as chronic leptin treatment in mice (225) or rats (226,227) has the ability to increase body temperature.

In contrast, most studies did not reveal an effect of leptin treatment on energy expenditure in *ad libitum*-fed wild-type mice (35,85,215).

## Leptin Can Prevent Torpor Bouts

As described, under conditions of nutrient/food deprivation, small mammals can enter short hibernation-like states, so-called torpor bouts. In this state, energy expenditure and body temperature both drop, thereby saving energy and securing survival when food is scarce. The induction of torpor has been linked to falling leptin levels in hamsters (83) and mice (49,79,85,90,228). *ob/ob* mice are more prone to induction of torpor than are lean mice, especially under conditions of starvation (49,78,79,90).

**Figure 8.**
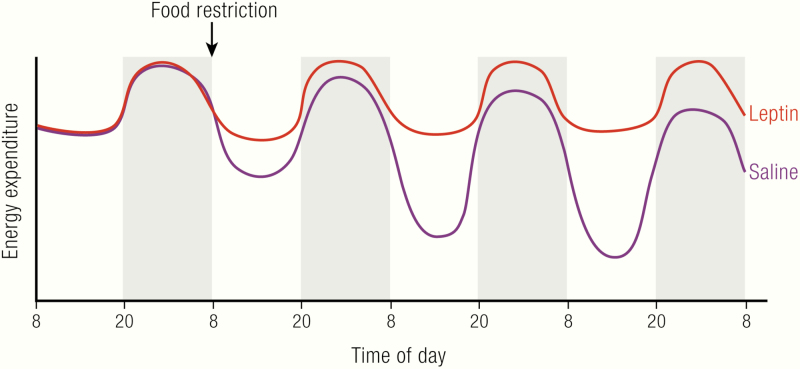
Leptin can prevent starvation-induced hypometabolic responses in lean mice. Although leptin treatment does not seem to induce any thermogenic response in lean or *ob/ob* mice under standard conditions, under some circumstances it can lead to elevated metabolic rates. Especially upon starvation or food restriction, body temperature and metabolism are usually decreased. Leptin treatment can prevent this drop in body temperature, resulting in higher energy expenditure. This is, however, not a classical thermogenic response (raising energy expenditure to produce heat), but rather an antitorpor effect of leptin (signalling to the brain that food reserves are sufficient to cope with the cold challenge).

Indeed, leptin replacement seems to be able to partially counteract the torpor induced by nutrient deprivation, thereby limiting the decrease in energy expenditure and body temperature (Fig. 8). This is mainly because of the prevention of torpor-induced reductions in metabolism (35,79,81,84–88), an effect that can also be mimicked by treating *ob/ob* mice with thyroid hormones (229) or an inhibitor of aspartate transcarbamylase, the rate-limiting enzyme of uridine synthesis (230). It is possibly this antitorpor effect of leptin that explains the findings of pair-feeding experiments, as discussed previously. The absence of leptin alone does not seem to be sufficient for torpor induction, but food deprivation is also needed to induce torpor.

## Leptin Effects on BAT in Lean Animals

Leptin has been reported to increase levels of UCP1 mRNA (88) or protein in rats (231) or to increase UCP1 mRNA in a NE-dependent manner in mice (232), while others have seen only minor or no effects in wild-type mice (53). GDP-binding was unaffected in *ad libitum*-fed leptin-treated rats (233). In contrast, Morrison (234,235) and Haynes et al. (231) found increased BAT sympathetic nerve activity in rats following intravenous leptin injections.

Some studies (236,237) report augmented reduction in fat weight in response to leptin treatment in the presence of UCP1 in pair-feeding experiments. However, also the extent of repression of food intake by leptin is decreased in the absence of UCP1 (236–238), which confounds the analysis of such pair-feeding experiments. The sympathetic innervation to BAT has been reported not to be necessary for the weight-reducing effects of leptin, also arguing against a strong role of BAT thermogenesis in the response to leptin (231). A positive effect on thermogenesis of increasing leptin sensitivity by decreasing leptin levels has been observed, paralleled by an increased locomotor activity (239).

## Central Administration of Leptin

Since leptin mainly acts via induction of signalling cascades in the hypothalamus, leading to nerve activation, direct injections of leptin into the ventricles (intracerebroventricular [ICV]) have been used repeatedly to identify the central pathways of leptin action. In clear contrast to the peripheral administration of leptin, which does not induce any BAT thermogenic program, the ICV means of administration seems to induce increases in energy expenditure in *ob/ob* mice and also in food-deprived lean mice (84). ICV injection of leptin has also been reported to elevate BAT temperature in wild-type mice (240), which, as stated, is not an accurate readout for BAT thermogenesis. Slightly elevated UCP1 mRNA levels in BAT of ICV leptin-injected mice and rats, as compared to saline-injected controls, have repeatedly been reported (88,240–242), and the increase in UCP1 expression is dependent on adrenergic signaling (243). General effects on sympathetic nerve activity following central administration of leptin have also been observed in other tissues (244), arguing against a specific effect on BAT sympathetic flow. Nevertheless, a study using an adeno-associated virus-mediated overexpression system that was injected ICV, reported increased UCP1 protein levels in BAT, an effect that was dependent on the sympathetic innervation of the tissue (231).

Nevertheless, the data obtained on thermogenesis induction in response to ICV leptin treatment are in clear contrast to the effects of peripheral treatment. Peripheral treatment does not result in increases in energy expenditure, whereas central administration clearly induced energy expenditure and presumably also sympathetic outflow to BAT. The differences between these 2 routes of administration are not easily understood, but it seems likely that high local concentrations of leptin in the hypothalamus may result in much stronger responses than peripheral injections. Although the effects of leptin are mainly centrally mediated, it remains to be determined whether the hypothalamic leptin concentrations induced by ICV injection resemble physiological states or whether this route of administration leads to artificially high responses that usually do not occur in a physiological context.

## Leptin in Humans

The discovery of leptin and its profound effects on body weight led to a search for humans with disturbances in leptin signalling. Indeed, leptin-deficient patients were found. However, while these patients displayed massive obesity (245), Farooqi et al. (246) found that the body temperature of a leptin-deficient child was normal and unaffected by leptin treatment. Although this may look different from data obtained in mouse studies, this child was most likely constantly under thermoneutral conditions, where *ob/ob* mice also display normal body temperature.

There are several reports of energy expenditure in leptin-deficient patients. Energy expenditure in leptin-deficient humans is similar to that in controls (247) or even higher, at least before adjustment for lean body mass differences (246). These findings thus seem to be in agreement with the data obtained in *ob/ob* mice at thermoneutrality, showing no difference in energy expenditure, or even higher energy expenditure.

Leptin treatment of leptin-deficient humans does not affect energy expenditure (248) or may slightly reduce energy expenditure, even when normalized to lean mass (246). However, others found that weight loss in leptin-deficient humans induced by diet restriction led to a reduction in energy expenditure. This did not happen when weight loss was achieved by leptin treatment (247).

It thus seems that leptin deficiency and leptin treatment do not affect energy expenditure or body temperature in humans under standard conditions. This is again in agreement with the data observed in mice, especially when experiments are performed at thermoneutrality. Also, in nonobese humans that do not display leptin deficiency, leptin treatment has been reported not to affect energy expenditure (249).

Weight loss and fasting are physiological states demonstrating low leptin levels. As stated earlier, leptin treatment of fasted wild-type mice can prevent the drop in body temperature and energy expenditure usually observed under these conditions. It may thus be speculated that similar effects should also be observed in humans. Indeed, several studies measured effects of leptin treatment on energy expenditure in humans during or after weight-loss, as summarized in (250) and outlined next.

In contrast to effects of leptin treatment in food-restricted mice, Fogteloo et al. (251) found no significant effect of leptin treatment in a study of obese humans during moderate caloric restriction. Others found that leptin was not able to prevent the drop in energy expenditure seen during caloric restriction in obese men (252) or during caloric restriction in overweight men (253,254).

In patients after weight loss, energy expenditure is significantly reduced (255). Several reports showed that leptin treatment can ameliorate or prevent the reductions in total energy expenditure in human cohorts after weight loss achieved by dietary restriction (256–258), while no effect was found in patients where weight loss had been achieved by Roux-en-Y gastric bypass surgery (259). Since also body temperature is reduced in humans after weight loss and caloric restriction (260–262), it seems reasonable to speculate that leptin, through its pyrexic effect, prevents a decrease in body temperature, thereby also affecting metabolism.

Taken together, the data on energy expenditure in humans suffering from leptin deficiency and humans receiving leptin treatment are remarkably similar to those obtained in mice. Leptin deficiency in humans does not seem to lead to reductions in energy expenditure or body temperature, just as it does not in *ob/ob* mice under thermoneutral conditions. Leptin treatment of these patients and of lean individuals does not affect energy expenditure, while in some states of physiological reduction of leptin levels, such as weight loss, leptin may prevent the drop in body temperature with the result that energy expenditure is increased.

## Conclusions

**Figure 9.**
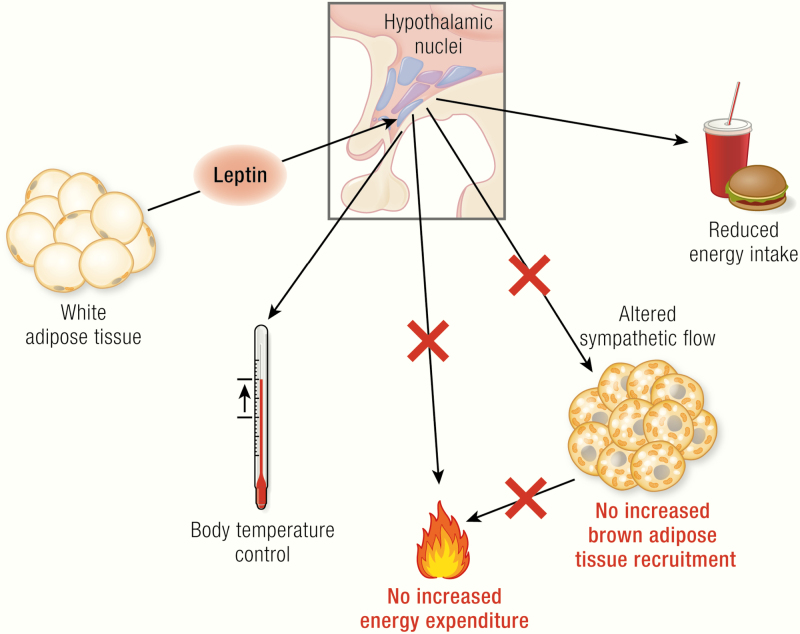
An updated picture of leptin action. Leptin acts in the brain to suppress food intake, and thereby leads to body weight reduction. Through its pyrexic effect, leptin controls body temperature by differentially regulating effector thresholds. However, and in contrast to the commonly accepted view, leptin has no thermogenic effect. It does not activate BAT-dependent thermogenesis, as is evident from the absence of an effect on energy expenditure. Leptin does not lead to thermogenic recruitment of BAT, but there are indications that leptin can affect sympathetic nerve growth and activity, thereby regulating lipolysis. Although leptin thus clearly affects food intake and body temperature thresholds, current experimental evidence neither supports an acute thermogenic effect of leptin, nor an effect on BAT thermogenic capacity. Leptin does, however, seem to affect the sympathetic innervation (and activity) in BAT and WAT, thereby affecting lipolysis and fuel supply, an effect calling for in-depth analysis in future studies.

The obese phenotype of the leptin-deficient *ob/ob* mouse has led to numerous investigations of the underlying cause of this dramatic phenotype. In addition to the irrefutable role of increased food intake in the development of their obesity, defects in energy metabolism, an inability to defend body temperature in cold environments, as well as atrophied BAT, have been held responsible for partially causing the adiposity in *ob/*ob mice (Fig. 1). However, published data on the effects of leptin deficiency on energy metabolism and BAT do not support these conclusions (Fig. 9).

Thus, in contrast to common understanding, leptin-deficient mice are not hypometabolic, but rather hypermetabolic under most conditions, an observation that can easily be overlooked when misleading normalization of energy expenditure is used. This is, however, in agreement with reports showing no difference or even higher energy expenditure in leptin-deficient humans. Under certain conditions, such as low leptin levels induced by weight loss or starvation, leptin replacement can, however, indeed increase energy expenditure in mice, rats, and humans. This is due to its ability to prevent reductions in body temperature and associated hypometabolic responses that usually occur in such situations.

Detailed examination of the literature on the role of leptin in the regulation of body temperature reveals that leptin-deficient *ob/ob* mice, rather than being unable to defend their body temperature, display a shift of the activation thresholds of thermoregulatory effectors toward lower temperatures, leading to a lower defended body temperature at subthermoneutral temperatures. This is rapidly corrected following leptin treatment because of the pyrexic effect of leptin, an effect that to some extent is also observed in wild-type mice. This response is, however, not associated with increases in thermogenesis or BAT recruitment, but rather mediated by decreases in heat loss.

Despite showing increased levels of lipid accumulation in BAT, BAT protein content and mitochondrial respiratory capacity are normal in most strains of leptin-deficient mice. UCP1 mRNA and protein levels, as well as the response to sympathomimetic drugs, are largely unaltered in *ob/ob* mice on the C57BL/6 background. Leptin-deficient mice on the outbred Aston background, as well as mice or rats lacking leptin receptors, show some signs of BAT atrophy as estimated from a slower response to cold. Most of these models display differences in GDP-binding and NE turnover; a phenotype presently not fully understood. Nevertheless, the quantitative significance of this apparent BAT atrophy for the development of obesity has yet to be established. Slow activation of BAT in response to cold exposure can probably not explain the acute cold intolerance of leptin-deficient mice because the acute response to cold mainly relies on shivering. A reduction in sympathetic activity in WAT may, however, result in impaired fuel supply for muscle shivering, thereby limiting shivering endurance and impairing the cold defense. We therefore think that the concept that leptin is thermogenic is probably misleading and has directed research efforts into less fruitful directions.

## Issues for Future Research

While leptin does not seem to affect energy expenditure, there are open questions that still need to be answered to fully understand the thermo-metabolic phenotype of the *ob/ob* mouse and the actions of leptin.

### Does torpor explain the pair-feeding results?

It appears that the only remaining argument supporting the existence of reductions in energy expenditure in *ob/ob* mice is based on results from pair-feeding experiments. However, initial experiments indicate induction of torpor during pair-feeding of *ob/ob* mice (35), an effect that is less pronounced in warmer environments. In agreement with this, the magnitude of the additional weight gain observed in pair-fed *ob/ob* mice decreases with increasing environmental temperature (91). The induction of torpor during pair-feeding may thus lead to reductions in energy expenditure in *ob/ob* mice, thereby apparently increasing their metabolic efficiency and lipid retention. The effect of pair-feeding and restricted feeding is influenced by the timing of the food supply during the light and dark phase, with the more physiologically relevant feeding time being the dark phase (263). Performing pair-feeding experiments at different environmental temperatures with continuous monitoring of body temperature during pair-feeding is warranted to identify the environmental temperature at which pair-feeding is not confounded by reductions in body temperature. It appears that this temperature may lie above 30°C (35) and only at this temperature would pair-feeding help to resolve the true effect of leptin deficiency on weight gain.

### Does BAT atrophy contribute to the obesity in Aston ob/ob mice?

A question remains as to which of the strains of the *ob/ob* displays the “real” phenotype of leptin-deficiency and whether BAT defects in *ob/ob* mice contribute in any way to their obesity (26). Especially on the outbred Aston background, the *ob/ob* mutation seems to result in some defects in BAT, and these mice also become more obese. Leptin replacement in these mice should therefore be performed to investigate if this reverses their phenotype and to exclude additional genetic alterations in these mice. Also, crossing the *ob* mutation into other outbred strains will help to delineate a direct effect of leptin on BAT physiology.

### Does poor sympathetic innervation explain the impaired cold response?

Rather than trying to find defects in BAT or thermoregulation in *ob/ob* mice in general, future research on leptin should aim at understanding the cause of the impaired response to acute cold exposure. A delay in both cold-induced BAT activation and probably also in the initiation of lipolysis providing fuel for shivering thermogenesis is one possible explanation. There seem to be differences in the sympathetic innervation of BAT, and also in sympathetically regulated lipolytic activity in WAT. Further, there are indications that, although BAT is fully functional in *ob/ob* mice, loss of UCP1 on the *ob/ob* background results in lethal hypothermia, even following gradual cooling (217), indicating that muscle shivering is impaired in *ob/ob* mice. Because leptin induces lipolysis in WAT through the sympathetic nervous system (164), measurement of lipolysis and fatty acid fluxes in *ob/ob* mice in response to cold stimulation (or exercise) may help in understanding the role of leptin in the regulation of whole body nutrient partitioning and shivering endurance.

### What is the molecular basis for the reduced sympathetic innervation?

Alterations in BAT (and WAT) sympathetic innervation, as well as delayed responses in GDP-binding and low NE turnover, indicate that the effects of leptin on BAT thermogenesis may lie not in the adipocytes themselves but rather in the sympathetic nerves. Leptin can directly stimulate axon outgrowth *in vitro* (161) and can induce alterations in dendritic morphology (264), but the molecular machinery through which leptin regulates nerve growth *in vivo* is presently not known. Mouse models, such as sympathetic nerve-specific LepR knockouts might help to identify direct, peripheral effects on nerve growth. Also, the effects of leptin on the development of the sympathetic nervous system and on the expression of factors known to regulate nerve growth, such as NGF, are interesting new research targets.

### How are the effects of leptin on body temperature mediated?

Leptin induces rapid changes in the defended body temperature without altering energy expenditure or behavioral body temperature regulation. Leptin administration leads to reductions in heat loss, presumably by increasing vasoconstriction, but whether these effects are centrally mediated or the result of peripheral effects of leptin on blood vessels remains to be clarified. However, direct leptin action is generally thought to rather induce vasodilation (265,266), whereas central leptin has been reported to affect vasoconstriction and blood pressure (244,267). The molecular regulation of this process and the role of leptin in integrating body temperature and heat loss remains to be determined. While some data point toward a role for leptin receptor-expressing neurons in the dorsomedial hypothalamus in mediating the effects of leptin on body temperature (225,268,269), the exact brain regions and neuronal populations mediating the effects of leptin on body temperature and the molecular mechanisms behind this are presently not identified.

## Abbreviations

BAT: brown adipose tissue
ICV: intracerebroventricular
NE: norepinephrine (= noradrenaline)
UCP1: uncoupling protein 1
WAT: white adipose tissue

## Additional Information

***Disclosure Summary:*** The authors have nothing to disclose.
